# Knowledge, Attitudes, and Practices concerning Black Fungus during COVID-19 Pandemic among Students of Bangladesh: An Online-Based Cross-Sectional Survey

**DOI:** 10.3390/ijerph19159146

**Published:** 2022-07-27

**Authors:** Md. Akhtarul Islam, Mst. Tanmin Nahar, Md. Nafiul Alam Khan, Zahid Ahmad Butt, A. S. M. Monjur-Al-Hossain, Sutapa Dey Barna, Md. Mostafizur Rahman, Henry Ratul Halder, Mohammed Zaber Hossain, Md. Tanvir Hossain

**Affiliations:** 1 Statistics Discipline, Science Engineering & Technology School, Khulna University, Khulna 9208, Bangladesh; akhtarulstat@ku.ac.bd (M.A.I.); tanminjemi@gmail.com (M.T.N.); nafiul.nipun95@gmail.com (M.N.A.K.); 2 School of Public Health Sciences, University of Waterloo, Waterloo, ON N2L 3G1, Canada; 3 Department of Pharmaceutical Technology, Faculty of Pharmacy, University of Dhaka, Dhaka 1000, Bangladesh; monjur.shiplu@du.ac.bd; 4 Department of Business Administration, International Standard University, 69 Mohakhali C/A, Dhaka 1212, Bangladesh; sutapa@isu.ac.bd; 5 Department of Disaster and Human Security Management, Faculty of Arts and Social Sciences, Bangladesh University of Professionals, Dhaka 1216, Bangladesh; mostafizur@bup.edu.bd; 6 Department of Community Health Sciences, Max Rady College of Medicine, University of Manitoba, Winnipeg, MB R3E 0W3, Canada; halderhr@myumanitoba.ca; 7 Department of English, Bangladesh Military Academy, Bhatiari, Chattogram 4315, Bangladesh; zaber5678@gmail.com; 8 Sociology Discipline, Social Science School, Khulna University, Khulna 9208, Bangladesh; tanvirku05@soc.ku.ac.bd

**Keywords:** Black Fungus, KAP analysis, COVID-19, students, Bangladesh

## Abstract

Background: Infection with COVID-19 and its control entailing steroids and immunomodulatory medications disrupted normal immune function. The ensuing immunological disorder led to the rise of another infection—Black Fungus (Mucormycosis). However, the spread of Black Fungus can be minimized through proper knowledge, informed attitude, and conscious preventive practice. This study aimed to assess students’ knowledge, attitude, and practice (KAP) regarding Black Fungus amid the COVID-19 pandemic in Bangladesh. Methods: This cross-sectional study was carried out among Bangladeshi students from June to July 2021. Using Google Forms, an e-questionnaire was developed for this web-based survey, and the participants were selected through a snowball sampling approach. Results: Out of the 2009 participants, more than half were female (53.5%), and the majority were at an age between 18 and 25 years (31.5%) and had received higher secondary (HSC) schooling (77.8%), while around 61% resided in urban areas. Findings revealed that most of the students (63.8%) spent around 2 h on electronic and social media to become informed about COVID-19 and Black Fungus. Approximately 33% of the students showed low KAP scores (32.9%), whereas around 26% had high KAP scores. Our results show a significant association between KAP and sex, schooling, living status, residence, and media exposure. Conclusion: The knowledge of Black Fungus considerably varies among Bangladeshi students considering the place of residence, age, sex, living arrangement, and media exposure. Policymakers should emphasize awareness among people focusing on the results of this study to increase deterrent attitudes and protective practices to minimize the risks of being infected.

## 1. Introduction

Following the outbreak of the novel coronavirus disease 2019 (COVID-19), also known as SARS-CoV-2, from Wuhan, China [1], the World Health Organization (WHO) declared COVID-19 a global pandemic on 11 March 2020 [2]. Since then, over 408 million people have been infected with COVID-19, while over 58 million have succumbed to death worldwide [3]. Likewise, the number of infected in Bangladesh was approximately 1.6 million, of which over 28 thousand had died of the infections as of 24 January 2022 [3,4].

The strict implementation of non-therapeutic measures [5,6] together with the quarantine and isolation of infected and suspected cases led to a decline in quality of life and aggravated psychological problems across different cohorts [7,8,9,10,11,12,13]. The intensified mental health issues further worsened with continuous exposure to “misinformation” through social and electronic media [14,15,16,17]. In addition, COVID-19 infection led to other consequences, especially in the Indian sub-continent, when another disease, *Mucormycosis*—a fungal infection, popularly known as Black Fungus—infected COVID-19 patients in India [18]. During the COVID-19 pandemic, Black Fungus infected around 7250 people in India [19]. Black Fungus, generally caused by a fungus in the order *Mucorales*, affects people, particularly diabetes patients, with a reduced ability to fight the infection, which helps the fungi to flourish more [19,20]. Although it is reported that Black Fungus affects 1.7 people per million, during the COVID-19 pandemic, the infection rate was eight times higher in India [21]. As of 2021, more than 40 thousand Black Fungus cases were reported, causing the death of over three thousand people [22,23]. Hence, the government of India declared Black Fungus as an epidemic according to the growing mortality rate [24].

Bangladesh—another South Asian country and a neighbour to India—reported its first two cases of Black Fungus on 8 May 2021, of which one patient died on 23 May 2021 [25]. Hence, Black Fungus has become a severe concern for patients that survived COVID-19 in Bangladesh [26,27]. Although Black Fungus does not spread from direct contact with infected people or animals [28], doctors often suggest removing the eyes of the infected to stop the spread of the virus to the brain, leading to mortality [29]. In such cases, immediate surgery is needed for more than 80% of the patients [30]. Henceforth, there is no alternative to early diagnosis of the disease, together with controlling diabetes and treating the patients with antifungal products, i.e., amphotericin B, and in most cases, surgical intervention, to minimize the risk factors [31]. Moreover, COVID-19 patients are advised not to expose themselves to any kind of natural habitats of the fungus [32]. In addition, the misuse of immunosuppressive medicine, comorbidities, and insalubrious preventive practices, such as the reuse of a “one-time mask” or an “unwashed mask”, were the most important influencing factors for COVID-19-related Black Fungus infections [25,33,34]. Inadequate knowledge of Black Fungus can also lead to inefficient control of the disease during this pandemic [35]. However, appropriate knowledge and awareness about Black Fungus could help minimize the spread of the disease during the ongoing pandemic [36,37], or else it could prove fatal for both COVID-19 patients and others recovering from the infection [25]. Currently, there are only a handful of studies that have assessed the knowledge and awareness of healthcare workers [38] and its possible health outcomes [39], but to the best of our knowledge, there is no study on the knowledge, attitude, and practice of students regarding Black Fungus and its associated factors in Bangladesh during the COVID-19 pandemic. Therefore, this study aimed to investigate students’ knowledge, attitude, and practice (KAP) regarding Black Fungus with different sociodemographic and COVID-19-related factors. It is important to note that students have greater access to the internet and higher exposure to information regarding COVID-19 and other associated risk factors through social and electronic media [15]; thereby, they are more vulnerable emotionally and mentally compared to other occupational groups and different cohorts [7,40,41]. Our objective was to assess the knowledge, attitude, and practice of Bangladeshi students with regards to Black Fungus during the COVID-19 pandemic. Therefore, students were selected to assess KAP regarding Black Fungus in order to minimize knowledge and awareness gaps and to reduce fear against Black Fungus among Bangladeshi students, their families, and peers. It is expected that the outcomes of this study may help policymakers take appropriate measures and implement remedial actions to improve the overall knowledge and awareness of students against Black Fungus in Bangladesh.

## 2. Materials and Methods

### 2.1. Study Design

This cross-sectional web-based survey was conducted among Bangladeshi students from June to July 2021. The countrywide lockdown and “social distancing” during the second wave of COVID-19 made it impossible to conduct a nationwide face-to-face survey; therefore, a web-based survey was conducted to reach out to students in Bangladesh. Moreover, a web-based survey during an infectious disease has already been proven as an efficient and effective way to understand the social dynamics of different infectious diseases [22,41,42,43,44].

### 2.2. Participants and Sampling

For this study, a snowball sampling approach was followed, and the participants were selected based on some specifications: (i) the participant must be a student; (ii) must have an active account on social media, e.g., Facebook, WhatsApp, Messenger, Viber; (iii) must have a capacity to read and understand the e-questionnaire written in English; (iv) must be a Bangladeshi citizen; and (v) stayed in Bangladesh during the COVID-19 outbreak. Considering the criteria mentioned above, the researchers communicated with teachers at different schools, colleges, and universities through email as well as social media, including Facebook, Messenger, and WhatsApp. The teachers were requested to forward the e-questionnaire to their students in Bangladesh. The student participants were further requested to forward the e-questionnaire to their school/college/university classmates and other students connected through different social media platforms. The data were collected for a month, from June 2021 to July 2021. From an initial response of over 2150, a total of 2009 responses were retained for this study, as some of the participants did not complete the e-questionnaire correctly, while there were some others who did not fulfill the criteria set by the researchers.

### 2.3. Ethical Approval

This research was ethically approved by the Khulna University Ethical Clearance Committee (Reference No. KUECC-2021/06/24). The e-questionnaire contained a consent form detailing the rights of the participants, i.e., the anonymity and confidentiality of the participants and their responses, respectively, and a right to withdraw without justification within a stipulated timeframe.

### 2.4. Survey Instrument

An e-questionnaire in English was developed for this web-based survey after a careful review of relevant studies [45,46,47,48]. The e-questionnaire was divided into five interrelated but distinct modules: Module 1 contained socioeconomic background (i.e., age, sex, religion, education, residence, marital status, living arrangement, media exposure); Module 2 highlighted health status (i.e., health status, COVID-19 status) of the participants; Module 3, Module 4, and Module 5 included questions on knowledge (8 items), attitude (5 items), and practice (5 items), respectively. The knowledge module concerning Black Fungus had questions about signs, symptoms, risk of infection, people at risk, and transmission. The attitude module emphasized a clean environment, measures against disease, and rumors. The practice module had questions mainly on the hygienic practices maintained by the participants. Each question had a three-point Likert-scale option, i.e., “disagree”, “neutral”, and “agree” options. Later, the responses were re-coded as 1 for accurate/correct answers and 0 for inaccurate/incorrect answers (including neutral responses) for analysis. The consistency test suggested that the three-point Likert-scale questions had acceptable reliability (Cronbach’s standard α value was in an accepted range of 0.60 to 0.84). For giving a correct answer, we provided a 1; for incorrect or neutral answers, we provided a 0. We then computed a new variable to re-categorize the old variable as a low, moderate, or high score. It is important to note that, for the new variable, a quartile term was followed to classify the score in which up to the 25th quartile was measured as a low score, 26th to 75th quartile was considered a moderate score, and greater than the 76th quartile was estimated as a high score in every section, namely, knowledge, attitude, and practice.

### 2.5. Statistical Data Analysis

Data were analyzed using the Statistical Package for the Social Sciences (SPSS) version 27 and STATA (version 14.2) computer software. A Chi-squared test was carried out to investigate the association between sociodemographic characteristics and knowledge, attitude, practice, and total KAP scores regarding Black Fungus. Shapiro–Wilk and Kolmogorov–Smirnov tests were conducted to determine the normality of numeric data about the total knowledge, attitude, practice, and total KAP scores. As the data were not normally distributed, therefore, non-parametric tests, such as the Kruskal–Wallis statistic test or Mann–Whitney U tests, were conducted to analyze the association between sociodemographic characteristics and knowledge, attitude, practice, and total KAP scores. A post hoc analysis was also executed through Bonferroni and Scheffé corrections for adjusting the significance value. In addition, descriptive statistics (frequency, percentage, median, and interquartile range) were also estimated. Finally, we utilized the generalized, ordered logistic regression model for predicting the knowledge, attitude, and practice level of students in Bangladesh [49]. For all statistical analyses, the α level (significance) was 0.05.

## 3. Results

### 3.1. Sociodemographic Outlines

Table 1 displays the sociodemographic characteristics of the participants. Among the participants, more than half were female (53.5%). The majority of the participants were from urban areas (60.7%), and most lived with their families (95.2%). Most of them (77.8%) received up to twelve years of schooling. About 31.5% of the study participants were in the age range of 18 to 25 years, and most of them (63.8%) spent less than or equal to 2 h daily on electronic and social media to become informed about COVID-19 and Black Fungus.

### 3.2. The Information Source of KAP Analysis towards Black Fungus

Table 2 shows the participants’ knowledge, attitude, and practice (KAP) towards Black Fungus. Most of the participants (74.4%) selected accurate responses in the statement about Black Fungus infection (usually infects the lung, sinus, stomach, and skin of a human). About 66% of the participants agreed with the factual statements about Black Fungus symptoms (headache, fever, cough, nausea, chest pain, vomiting, and breathlessness). More than half (55.3%) of the participants precisely knew that the spread of Black Fungus could be minimized by universal masking.

Most participants (80.9%) reported that keeping updated with the current situation is essential for individual and collective well-being from Black Fungus; 77.3% of the students stated that the information shared by government agencies is vital to control the disease and rumors. Table 2 also shows the practices of the participants regarding Black Fungus. The majority of the participants (89.1%) reported good practices to protect themselves against Black Fungus and COVID-19 by wearing facemasks if they went to the market or other crowded places. However, many (81.5%) revealed that they tried to avoid areas covered with dust, such as construction areas, which are the critical paths of receiving fungal spore transmission from the environment to the human body. The remaining inquiries concerning KAP towards Black Fungus and answers are presented in Table 2.

### 3.3. Knowledge, Attitude, Practice, and Total KAP of the Students

Table 3 indicates the associations between different sociodemographic factors with knowledge scores (low, moderate, high) concerning Black Fungus. Suggestively, many participating students’ knowledge scores on Black Fungus were shown to be significantly related to their sex identity, religious identity, living status, health status, history of COVID-19, media exposure, source of information, time spent (hours) on media, and exposure to media compared to pre-COVID-19 situation (considered *p* > 0.05 for all). Most participants demonstrated a moderate knowledge score (41.3%) and a low knowledge score (38.3%). Female students showed relatively better knowledge; 23.7% had moderate knowledge, while 12% had high knowledge, compared to their male equivalents (17.2% moderate and 8.2% high knowledge score). Regarding religion, Muslim students showed moderate (38.6%) knowledge about Black Fungus. Students living with families—with or without children—also had better knowledge about Black Fungus (19.9% high and 39.3% moderate knowledge). In addition, students with greater exposure to information through radio or television had high knowledge (10.0%) score, and exposure from other sources of information, such as social media, exhibited a high (7.6%) knowledge score on Black Fungus. Again, Table 3 indicates the association between sociodemographic factors with attitude scores (low, moderate, and high) concerning Black Fungus. Many participating students’ attitude scores on Black Fungus were shown to be significantly related to their sex identity, religious identity, schooling, marital status, living status, living location, media exposure, source of information, and exposure to media compared to pre-COVID-19 situation (considered *p* > 0.05 for all). Findings suggest that most of the students showed low (40.8%) to moderate (50.8%) attitude scores. Female students (29.4%) and those living with families (49.0%) and in urban areas (32.0%) attained moderate attitude scores compared to male students (21.1%) and those living away from families (1.8%) and in rural or suburban areas (18.8%). It was also found that students with greater exposure to information through radio and television showed moderate (24.5%) attitude to high (4.4%) attitude scores regarding Black Fungus.

Table 3 specifies the association between sociodemographic features and practice scores (low, moderate, and high) concerning Black Fungus. It was observed that many participants’ practice scores on Black Fungus were shown to be significantly related to their sex identity, age (years), schooling, living status, living location, health status, history of COVID-19, media exposure, source of information, time spent (hours) on media, and exposure to media compared to pre-COVID-19 situation (considered *p* > 0.05 for all). A quarter of the students (25%) were reluctant to take preventive measures to reduce the risk of Black Fungus infection, whereas 38.4% took all possible measures and scored higher regarding practice to prevent Black Fungus infection. It is apparent that female students (23.3%), as well as those living in urban areas (24.7%) and with families (37%), took necessary actions to reduce the risk of Black Fungus infection compared to male students and those living in rural or suburban areas (13.7%) and living alone (1.4%). It is also observed that older students were relatively unwilling to follow the non-therapeutic measures compared to younger students. In addition, students with better exposure to information, mainly through radio and television (20.2%), were more interested in following protective measures. Moreover, Table 3 illustrates the association between sociodemographic factors and total KAP score (low, moderate, and high) regarding Black Fungus. Many students achieved low KAP scores (32.9%); however, 25.7% obtained high KAP scores. Findings indicate that students living with families (25%) and in urban areas (16.4%) showed higher KAP scores than students living alone and in rural or suburban areas. Likewise, female students (15.7%) and those with education up to higher secondary level (21%) outperformed regarding high KAP scores when compared to male students (9.9%) and students with an undergraduate degree (1.4), respectively. Furthermore, students with greater exposure to information through radio and television showed higher KAP scores (13.4%).

Table 4 summarizes the association of the KAP score regarding Black Fungus with sociodemographic background. Post hoc analysis showed that female students demonstrated significantly higher knowledge, attitude, practice, and total KAP scores than males. Considering the religious identity, Muslim students showed higher knowledge, practice, and total KAP scores when compared to students from other religious groups. Unlike older students (18–25 years), younger students (≤17 years) showed significantly higher attitude, practice, and total KAP scores. Likewise, students who had schooling up to HSC reportedly had higher attitude, practice, and total KAP scores than students with undergraduate and graduate degrees. Unmarried students had significantly better attitudes and total KAP scores towards Black Fungus than ever-married students. Regarding living arrangements, students living with families had significantly higher knowledge, attitude, practice, and total KAP scores when compared to students living alone. Urban areas students showed significantly better attitude, practice, and total KAP scores than their rural counterparts.

Post hoc analysis further showed that students with good and excellent health status exhibited significantly better scores than students with poor health status. Moreover, students not affected by COVID-19 had higher scores in KAP than those infected or at risk of infection with COVID-19. Students with greater media exposure had significantly higher knowledge, practice, and total KAP scores than students with low or no media exposure. Moreover, students who received information from radio and television reportedly had significantly higher attitude, practice, and total KAP scores than students receiving news from social media and other internet sources. Students who spent less time (≤2 h) seeking information showed significantly higher attitude, practice, and total KAP scores than those who spent more than five hours a day. Increased exposure to media compared to pre-COVID-19 situation among students demonstrated significantly higher scores in all sections than the students with decreased exposure to media from pre-COVID-19.

Table 5 shows the generalized, ordered logistic regression model of knowledge, attitude, and practice level of the students concerning Black Fungus with adjusted odds ratio and 95% confidence level. A significant generalized, ordered logistic regression was found for knowledge level (LR χ2 = 131.92), attitude level (LR χ2 = 154.53), and practice level (LR χ2 = 224.06) at 1% level of significance.

In this model, sex identity, living location, history of COVID-19, source of information, time spent (hours) on media, and media exposure were found to be significantly associated with knowledge level among students during the COVID-19 pandemic towards Black Fungus. Compared to male students, female students were 1.49 times (aOR: 1.49, 95% CI = 1.23, 1.81) more likely to be in the moderate and higher knowledge group for Black Fungus than the lower knowledge group. In the case of the higher knowledge group compared to the lower and moderate knowledge group, the female students were 1.27 times (aOR: 1.27, 95% CI = 1.01, 1.60) more likely to be in the higher knowledge group for Black Fungus compared to male students. When compared to students who never used media to gather information, students who always used media were 2.05 times (aOR: 2.05, 95% CI = 1.41, 2.97) more likely to be in the moderate and higher knowledge group for Black Fungus than the lower knowledge group. Similarly, comparing the students who never used media, the observed knowledge for the students who always used media to obtain information about Black Fungus was 2.11 times more (aOR: 2.11, 95% CI = 1.29, 3.47) likely to be in the higher knowledge group than in the lower and moderate knowledge group.

Again, considering attitude as a dependent variable, the sex identity, religious identity, age (years), marital status, media exposure, source of information, and exposure to media compare to pre-COVID-19 were found to be significantly associated with attitude levels among students during the COVID-19 pandemic towards Black Fungus. Compared to male students, female students were 1.58 times (aOR: 1.58, 95% CI = 1.30, 1.91) more likely to have a higher and moderate attitude level than a lower attitude level. Students belonging to the Hindu religion were 1.55 times more likely (aOR: 1.55, 95% CI = 1.04, 2.29) to be in the higher and moderate attitude group than in the lower attitude group compared to Muslim students concerning Black Fungus. Similarly, compared to the lower attitude group students, students in the ≥26 age group were 2.21 times more (aOR: 2.21, 95% CI = 1.15, 4.04) likely be to in the higher and moderate level regarding Black Fungus compared to those who were in ≤17 age group.

Students who acquired their information from YouTube and online news were 2.39 times more (aOR: 2.39, 95% CI = 1.85, 3.85) likely to be in the higher attitude group versus lower and moderate attitude than those who receive their information from social media. Students who had the same exposure to media compared to pre-COVID-19 situation were 2.39 times more (aOR: 2, 39, 95% CI = 1.00, 5.72) likely to exist in the higher attitude group compared to those students who had decreased exposure to COVID-19 concerning Black Fungus.

Moreover, in the model with the dependent variable practice, we found that sex identity, age (years), living location, history of COVID-19, media exposure, source of information, time spent (hours) on media, and exposure to media compared to pre-COVID-19 situation were found significantly associated with practice level among students during the COVID-19 pandemic towards Black Fungus. It shows that female students were 1.57 times (aOR: 1.57, 95% CI = 1.29, 1.91) more likely to be in the higher practice group than those in the lower and moderate practice group concerning Black Fungus than male students. Compared to the students in ≤17 age group, students in the ≥26 age group are 3.13 times more (aOR: 3.13, 95% CI = 1.55, 6.33) likely to be in the higher practice level compared to the lower and moderate practice level regarding Black Fungus. Compared to the students who never used to obtain information from media, students who always used media were 3.80 times (aOR: 3.80, 95% CI = 2.57, 5.66) more likely to be in the moderate and higher practice group than in the lower practice group. Moreover, students who acquired their information from radio and television were 1.39 times (aOR: 1.39, 95% CI = 1.08, 1.79) more likely to be in the moderate and higher practice group than those who receive their information from social media. Furthermore, comparing the students who had decreased exposure to COVID-19 concerning Black Fungus, students who had increased exposure to COVID-19 were 1.56 times more (aOR: 1.56, 95% CI = 1.06, 2.31) likely to be in the higher practice group than in the lower and moderate practice group.

## 4. Discussion

This study assessed students’ knowledge, attitude, and practice regarding Black Fungus. It is noteworthy that no previous research on KAP analysis of Black Fungus has been conducted in Bangladeshi students. Our results indicate that six out of ten students (61.7%) had proper knowledge (moderate 41.3%, high 20.4%) scores about Black Fungus, implying that nearly 40% of Bangladeshi students (38.3%) had low to no knowledge about Black Fungus. The knowledge score (61.2%) in a prior study of young adults in Bangladesh regarding COVID-19 [49] was almost equivalent to the knowledge score of the current study (61.7%). However, a study on the knowledge score of COVID-19 among Chinese students (90%) was significantly higher than our current study on the knowledge score of Black Fungus among Bangladeshi students [50].

In addition, our study also found that sex identity, living, history of COVID-19, media exposure, source of information, and time spent on social media were all significantly linked to the knowledge score of students regarding Black Fungus. Our findings corroborate that of a prior COVID-19 study in Bangladesh [51]. It is evident that greater access to and use of the internet and relevant technology by the younger population, especially students, could have contributed to their higher knowledge. Studies suggest that the use of media and technology has increased during the lockdown among the younger population [52,53,54,55]. Furthermore, our results indicate that students’ knowledge regarding Black Fungus in Bangladesh was significantly influenced by their personal characteristics, such as gender, religion, residence, and health status. Previous studies showed that knowledge about any pandemic varies according to gender and marital status, which matches with our study [56,57]. Our study found that having been infected with COVID-19 was significantly linked to growing knowledge about Black Fungus. It is, however, essential to note that the growing death rate from Black Fungus in the COVID-19 era [58] led to an increasing fear among people, largely due to a lack of proper knowledge about Black Fungus [59].

Similar to the knowledge score, this study’s findings showed a significant association of sex identity, religious identity, age, marital and living status, history of COVID-19, media exposure, source of information, time spent on media, and exposure to media compared to pre-COVID-19 situation with the attitude score of the students regarding Black Fungus. Previous studies show similar results that age, gender, media exposure, and marital status have a significant association with the attitude score of pandemic diseases [48,60,61]. Furthermore, participants were more likely to have an optimistic attitude and understand the disease from different sources than those with poor knowledge [60]. Similarly, prior research indicated that participants’ positive attitude was significantly correlated with knowledge of the diseases [62,63]. On the other hand, a study from Pakistan showed that the participants’ attitudes were not influenced by age, job or occupation, or gender [64].

This study found that sex identity, religion, age, and media exposure were significantly correlated with a positive attitude, and the majority of participants (80.9%) were aware of the risk of Black Fungus. Our findings also revealed that approximately 77.3% of the students believed that information provided by government agencies is critical to controlling the disease. This study discovered that a higher proportion of female students had a more positive (29.4%) attitude than their male equivalents (21.1%). Likewise, it was reported that students who had more exposure to information via radio and television had attitudes toward Black Fungus that ranged from moderate (24.5%) to high (24.5%). On the other hand, a study in Thailand showed that attitudes were subject to gender and age [65]. Again, the present study indicated that most students (89.1%) reported using protective practices against Black Fungus, including wearing a facemask or avoiding markets or other crowded places. Our study showed that Bangladeshi students were more aware of non-therapeutic practices than knowledge and attitudes toward Black Fungus, which was similar to a study conducted in 2021 about the attitude of Bangladesh university students towards COVID-19 [47]. Another survey of COVID-19 in Bangladesh, in contrast, found that many Bangladeshi youths (48.4%) did not take any preventive actions against COVID-19 [50]. In addition, the results of our study indicate that there exists a significant association of sex identity, history of COVID-19, media exposure, source of information, time spent on media, age, and exposure to media compared to pre-COVID-19 situation with the practice score of the students regarding Black Fungus. Results from previous studies showed that additional frequent practices of any pandemic disease were significantly associated with participants’ age and sex, occupation, marital status, and place of residence, which support the results of our study [48,66,67]. Studies on infectious diseases suggest that females outperform males in knowledge, attitudes, and practices regarding contagious diseases (e.g., H1N1, SARS, etc.) [68,69]. The sex differences were also demonstrated in the current investigation, as it is evident that female students had a higher score in the KAP total score (15.7%) than men (9.9%) did. According to the findings, many of the students who took part in the study had low KAP scores (32.9%), while 25.7% had high KAP scores. Male students receiving up to higher secondary education, living alone or with friends, residing in rural or suburban areas, and obtaining information from a different source than radio or television were associated with an inappropriate KAP score.

At the beginning of COVID-19, an extensive amount of misinformation spread across the world via modern technologies, and students were the main victims of this misinformation. It is crucial to investigate the current knowledge, attitude, and practice of the students toward Black Fungus in order to guide policymakers and public health professionals in ways in which the best outcomes can be achieved through public health interventions. To prevent the further rise in Black Fungus incidence and potential health hazards where early diagnosis is essential in accomplishing a positive outcome, proper knowledge among students is a must.

### Limitations and Strengths

This study is the first to investigate students’ knowledge, attitude, and practice regarding Black Fungus (Mucormycosis) amid the COVID-19 pandemic in Bangladesh. This research used a web-based investigation to maintain “social distancing” and to avoid the risk of infection—a universalized and validated approach to carry out research during an outbreak of infectious disease. Some limitations may apply to this research. Considering that this study was cross-sectional, it may be challenging to draw any strong conclusions about causality. Self-reporting has several drawbacks, such as different biases, in comparison to face-to-face interviews. The COVID-19 pandemic has shown how inadequately equipped the Least Developed Countries (LDCs) are for the technological environment despite the Sustainable Development Goal (SDG) to increase access to communications technology and reasonable access to the internet in developing countries by 2020, which has not been fulfilled yet. Consequently, this study failed to ensure a representative student sample, as this study only collected data from those with internet access in Bangladesh. We utilized a restricted number of questions to assess knowledge, attitude, and practice. Thus, extensive and inclusive studies would be necessary to ascertain the true prevalence of KAP regarding Black Fungus among the general population.

## 5. Conclusions

The findings of this study suggest that knowledge, attitude, and practice toward Black Fungus significantly differs among Bangladeshi students with regards to sex, living arrangement, age, places of residence, and media exposure. It indicates that we could control and anticipate Black Fungus and relevant infectious diseases by maintaining safe practices and encouraging favorable attitudes with the cooperation of the authorities, health policymakers, and the general population. This study suggests maintaining a precautionary behavior to increase safe practices toward Black Fungus. Specific interventions need to be designed and implemented for the students who live in rural areas to increase their knowledge and awareness about Black Fungus. Increased media exposure is required to reach male students as well as respondents who are living in rural areas. Critical and timely insights from this research may also guide policymakers in improving strategic planning for COVID-19 and Black Fungus outbreaks in developing countries, including Bangladesh.

## Figures and Tables

**Table 1:** Sociodemographic characteristics of study participants (*N* = 2009).

Variables	Frequency (*f*)	Percentage (%)
**Sex identity**		
Male	914	45.5
Female	1074	53.5
Prefer not to say	21	1.0
**Religious identity**		
Muslim	1842	91.7
Hindu	129	6.4
Others	38	1.9
**Age (years)**		
≤17	1275	63.5
18–25	632	31.5
≥26	102	5.0
**Schooling**		
Up to HSC	1562	77.8
Honors	314	15.6
Masters and above	133	6.6
**Marital status**		
Never married	1805	89.8
Ever married	204	10.2
**Living status**		
With family (including children)	1912	95.2
Alone or with friends	97	4.8
**Living location**		
Rural and Suburban	789	39.3
Urban	1220	60.7
**Health status**		
Poor	34	1.7
Fair	107	5.3
Good	867	43.2
Very good	567	28.2
Excellent	434	21.6
**Ever COVID-19 affected**		
No	1590	79.2
Maybe/not sure	332	16.5
Yes	87	4.3
**Media exposure**		
Never	155	7.7
Occasionally/sometimes/often	1291	64.2
Always	564	28.1
**Source of information**		
Social media	744	37.0
YouTube and online news	358	17.8
Radio and television	907	45.2
**Time spent (hours) on media**		
≤2 h	1282	63.8
3–4 h	472	23.5
5 h and above	255	12.7
**Exposure to media compared to pre-COVID-19 situation**		
Decreased	150	7.5
About the same	590	29.4
Increased	1269	63.1

**Table 2:** Knowledge, attitude, and practice (KAP) of participants towards Black Fungus (*N* = 2009).

Questions/Statements		True *n* (%)	False *n* (%)
**Knowledge**			
1	Black Fungus is contagious.	1093 (54.4)	916 (45.6)
2	The Black Fungus spreads through breathing fungal spores from the environment.	1308 (65.1)	701 (34.9)
3	Black Fungus usually infects the lung, sinus, stomach, and skin of a human.	1495 (74.4)	514 (25.6)
4	The symptoms of Black Fungus consist of headache, fever, cough, nausea, chest pain, vomiting, and breathlessness.	1325 (66.0)	684 (34.0)
5	There is a test to confirm a Black Fungus or a vaccine to prevent it.	503 (25.0)	1506 (75.0)
6	Most patients infected with Black Fungus are those who recovered from the COVID-19.	1244 (61.9)	765 (38.1)
7	The spread of Black Fungus can be minimized by universal masking.	1111 (55.3)	898 (44.7)
8	Elderly and chronic patients are more vulnerable to Black fungus.	1436 (71.5)	573 (28.5)
**Attitude**			
1	Keeping updated with the current situation of Black Fungus is essential for individual and collective well-being.	1625 (80.9)	384 (19.1)
2	I felt worried/scared after knowing the growing number of deaths across the world due to the Black Fungus.	1468 (73.1)	541 (26.9)
3	The information shared by the Government agencies is essential to control the disease and rumors.	1552 (77.3)	457 (22.7)
4	People infected with or suspected of Black Fungus should be labeled or stigmatized.	361 (18)	1648 (82.0)
5	Emphasis should be placed on keeping the environment clean and maintaining personal hygiene.	1735 (86.4)	274 (13.6)
**Practice**			
1	I try to avoid areas covered with dust, like construction sites.	1637 (81.5)	372 (18.5)
2	I wear a facemask if I go to the market or other crowded places.	1790 (89.1)	219 (10.9)
3	I try to avoid visiting water-damaged buildings and flooded areas.	1513 (75.3)	496 (24.7)
4	I do not wear shoes, gloves, masks, long pants, and a long-sleeve shirt while getting in contact with soil and dust.	1195 (59.5)	814 (40.5)
5	I clean skin injuries with disinfectants, especially after exposure to soil or dust.	1663 (82.8)	346 (17.2)

**Table 3:** Factors associated with the knowledge score, attitude score, practice score, and total KAP score concerning Black Fungus.

Factors	Knowledge Score				Attitude Score				Practice Score				Total KAP Score			
	Low Score *n* (%)	Moderate Score *n* (%)	High Score *n* (%)	*p*-Value	Low Score *n* (%)	Moderate Score *n* (%)	High Score *n* (%)	*p*-Value	Low Score *n* (%)	Moderate Score *n* (%)	High Score *n* (%)	*p*-Value	Low Score *n* (%)	Moderate Score *n* (%)	High Score *n* (%)	*p*-Value
Knowledge score	770 (38.3)	830 (41.3)	409 (20.4)		819 (40.8)	1021 (50.8)	169 (8.4)		502 (25.0)	735 (36.6)	772 (38.4)		660 (32.9)	832 (41.4)	517 (25.7)	
Sex identity																
Male	402 (20.0)	345 (17.2)	167 (8.3)	0.000	429 (21.4)	424 (21.1)	61 (3.0)	0.000	271 (13.5)	343 (17.1)	300 (14.9)	0.000	361 (18.0)	354 (17.6)	199 (9.9)	0.000
Female	357 (17.8)	476 (23.7)	241 (12.0)		377 (18.8)	590 (29.5)	107 (5.3)		218 (10.9)	388 (19.3)	468 (23.3)		287 (14.3)	472 (23.5)	315 (15.7)	
Prefer not to say	11 (0.5)	9 (0.4)	1 (0.0)		13 (0.6)	7 (0.3)	1 (0.0)		13 (0.6)	4 (0.2)	4 (0.2)		12 (0.6)	6 (0.3)	3 (0.1)	
Religious identity																
Muslim	690 (34.4)	775 (38.6)	377 (18.8)	0.039	756 (37.7)	931 (46.4)	155 (7.7)	0.031	448 (22.3)	681 (33.9)	713 (35.6)	0.147	593 (29.5)	771 (38.4)	478 (23.9)	0.088
Hindu	59 (2.9)	42 (2.1)	28 (1.4)		42 (2.1)	78 (3.9)	9 (0.4)		39 (1.9)	44 (2.2)	46 (2.3)		47 (2.3)	49 (2.4)	33 (1.6)	
Others	21 (1.0)	13 (0.6)	4 (0.2)		21 (1.0)	12 (0.6)	5 (0.2)		15 (0.7)	10 (0.5)	13 (0.6)		20 (1.0)	12 (0.6)	6 (0.3)	
Age (years)																
≤17	472 (23.5)	537 (26.8)	266 (13.2)	0.621	500 (24.9)	657 (32.7)	118 (5.9)	0.226	303 (15.1)	440 (21.9)	532 (26.5)	0.000	399 (19.9)	523 (26.0)	353 (17.7)	0.049
18–25	257 (12.8)	251 (12.5)	124 (6.2)		277 (13.8)	312 (15.5)	43 (2.1)		176 (8.8)	261 (13.0)	195 (9.7)		230 (11.4)	261 (13.0)	141 (7.0)	
≥26	41 (2.0)	42 (2.1)	19 (0.9)		42 (2.1)	52 (2.6)	8 (0.4)		23 (1.1)	34 (1.7)	45 (2.2)		31 (1.5)	48 (2.4)	23 (1.1)	
Schooling																
Up to HSC	572 (28.5)	660 (32.9)	330 (16.4)	0.063	605 (30.1)	819 (40.8)	138 (6.9)	0.012	368 (18.3)	555 (27.6)	639 (31.9)	0.000	477 (23.7)	664 (33.1)	421 (21.0)	0.001
Honors	140 (7.0)	119 (5.9)	55 (2.7)		153 (7.6)	138 (6.9)	23 (1.1)		97 (4.8)	130 (6.5)	87 (4.3)		132 (6.6)	114 (5.6)	68 (3.4)	
Masters and above	58 (2.9)	51 (2.5)	24 (1.2)		61 (3.0)	64 (3.2)	8 (0.4)		37 (1.8)	50 (2.5)	46 (2.3)		51 (2.5)	54 (2.7)	28 (1.4)	
Marital status																
Never married	687 (34.3)	744 (37.0)	374 (18.6)	0.470	719 (35.8)	934 (46.5)	152 (7.6)	0.034	446 (22.2)	655 (32.6)	704 (35.0)	0.285	588 (29.3)	741 (36.9)	479 (23.7)	0.152
Ever married	83 (4.1)	86 (4.3)	35 (1.7)		100 (5.0)	87 (4.3)	17 (0.8)		56 (2.8)	80 (4.0)	68 (3.4)		72 (3.6)	91 (4.5)	41 (2.0)	
Living status																
With family (including children)	723 (36.0)	789 (39.3)	400 (19.9)	0.012	765 (38.1)	984 (49.0)	163 (8.1)	0.009	465 (23.2)	704 (35.1)	743 (37.0)	0.008	617 (30.7)	792 (39.5)	503 (25.0)	0.010
Alone or with friends	47 (2.3)	41 (2.1)	9 (0.4)		54 (2.7)	37 (1.8)	6 (0.3)		37 (1.8)	31 (1.5)	29 (1.4)		43 (2.1)	40 (2.0)	14 (0.7)	
Living location																
Rural and Suburban	320 (15.9)	303 (15.1)	166 (8.3)	0.098	357 (17.8)	378 (18.8)	54 (2.7)	0.002	240 (11.9)	273 (13.6)	276 (13.7)	0.000	287 (14.3)	314 (15.6)	188 (9.4)	0.023
Urban	450 (22.4)	527 (26.2)	243 (12.1)		462 (23.0)	643 (32.0)	115 (5.7)		262 (13.0)	462 (23.0)	496 (24.8)		373 (18.6)	518 (25.7)	329 (16.4)	
Health status																
Poor	18 (0.9)	11 (0.6)	5 (0.2)	0.015	17 (0.8)	16 (0.8)	1 (0.0)	0.480	15 (0.7)	6 (0.3)	13 (0.6)	0.012	17 (0.8)	11 (0.5)	6 (0.3)	0.001
Fair	53 (2.6)	37 (1.8)	17 (0.9)		47 (2.3)	55 (2.7)	5 (0.2)		34 (1.7)	42 (2.1)	31 (1.5)		50 (2.5)	41 (2.0)	16 (0.8)	
Good	334 (16.6)	352 (17.5)	181 (9.0)		345 (17.3)	442 (22.0)	80 (4.0)		220 (11.0)	332 (16.5)	315 (15.7)		289 (14.5)	353 (17.7)	225 (11.2)	
Very good	204 (10.2)	262 (13.0)	101 (5.0)		220 (11.0)	299 (14.9)	48 (2.4)		132 (6.6)	209 (10.5)	226 (11.2)		166 (8.3)	262 (13.0)	139 (6.9)	
Excellent	161 (8.1)	168 (8.4)	105 (5.2)		190 (9.5)	209 (10.4)	35 (1.7)		101 (5.0)	146 (7.3)	187 (9.3)		138 (6.8)	165 (8.2)	131 (6.5)	
Ever COVID-19 affected																
No	581 (28.9)	669 (33.3)	340 (16.9)	0.019	630 (31.4)	823 (41.0)	137 (6.8)	0.154	375 (18.7)	567 (28.2)	648 (32.2)	0.001	488 (24.3)	662 (33.0)	440 (21.9)	0.000
Maybe/not sure	151 (7.5)	125 (6.2)	56 (2.8)		146 (7.3)	163 (8.1)	23 (1.1)		102 (5.1)	130 (6.5)	100 (5.0)		137 (6.8)	135 (6.7)	61 (3.0)	
Yes	38 (1.9)	36 (1.8)	13 (0.6)		43 (2.1)	35 (1.7)	9 (0.4)		25 (1.2)	38 (1.9)	24 (1.2)		36 (1.8)	35 (1.7)	16 (0.8)	
Media exposure																
Never	82 (4.1)	50 (2.5)	23 (1.1)	0.000	92 (4.6)	51 (2.5)	12 (0.6)	0.000	78 (3.9)	37 (1.8)	40 (2.0)	0.000	84 (4.2)	44 (2.2)	27 (1.3)	0.000
Occasionally/sometimes/often	497 (24.7)	551 (27.5)	242 (12.0)		526 (26.2)	664 (33.1)	100 (5.0)		316 (15.8)	473 (23.5)	501 (24.9)		433 (21.6)	548 (27.3)	309 (15.4)	
Always	191 (9.5)	229 (11.4)	144 (7.2)		201 (10.0)	306 (15.2)	57 (2.8)		108 (5.4)	225 (11.2)	231 (11.5)		143 (7.1)	240 (11.9)	181 (9.0)	
Source of information																
Social media	322 (16.0)	269 (13.4)	153 (7.6)	0.000	344 (17.1)	362 (18.0)	38 (1.9)	0.000	225 (11.2)	288 (14.3)	231 (11.5)	0.000	292 (14.5)	285 (14.2)	167 (8.3)	0.000
YouTube and online news	135 (6.7)	167 (8.3)	56 (2.8)		150 (7.5)	166 (8.3)	42 (2.1)		93 (4.6)	129 (6.4)	136 (6.8)		116 (5.8)	161 (8.0)	81 (4.0)	
Radio and television	313 (15.6)	394 (19.6)	200 (10.0)		325 (16.2)	493 (24.5)	89 (4.4)		184 (9.2)	318 (15.8)	405 (20.2)		252 (12.5)	386 (19.2)	269 (13.5)	
Time spent (hours) on media																
≤2 h	478 (23.8)	552 (27.6)	252 (12.5)	0.003	507 (25.2)	656 (32.7)	119 (5.9)	0.132	277 (13.8)	480 (23.9)	525 (26.1)	0.000	392 (19.5)	553 (27.6)	337 (16.8)	0.000
3–4 h	169 (8.4)	199 (9.9)	104 (5.2)		202 (10.1)	232 (11.5)	38 (1.9)		132 (6.6)	156 (7.8)	184 (9.2)		151 (7.5)	196 (9.8)	125 (6.2)	
5 h and above	123 (6.1)	79 (3.9)	53 (2.6)		110 (5.5)	133 (6.6)	12 (0.6)		93 (4.6)	99 (4.9)	63 (3.1)		117 (5.8)	83 (4.1)	55 (2.7)	
Exposure to media compare to pre COVID-19 situation																
Decreased	68 (3.4)	48 (2.4)	34 (1.7)	0.040	77 (3.8)	67 (3.3)	6 (0.3)	0.001	57 (2.8)	53 (2.6)	40 (2.0)	0.002	73 (3.6)	46 (2.3)	31 (1.5)	0.000
About the same	242 (12.0)	239 (11.9)	109 (5.4)		263 (13.1)	273 (13.6)	54 (2.7)		145 (7.2)	220 (11.0)	225 (11.2)		205 (10.2)	238 (11.8)	147 (7.3)	
Increased	460 (22.9)	543 (27.1)	266 (13.2)		479 (23.9)	681 (33.9)	109 (5.4)		300 (14.9)	462 (23.0)	507 (25.2)		382 (19.1)	548 (27.3)	339 (16.9)	

**Table 4:** Assessment of total knowledge, attitude, practice, and total KAP scores concerning Black Fungus.

Factors	Knowledge (Median (IQR))	Attitude (Median (IQR))	Practice (Median (IQR))	Total KAP (Median (IQR))
**Sex identity**				
Male	5 (3) ***	4 (1) *	4 (2) *	12 (4) *
Female	**5 (2)**	**4 (1)**	**4 (1)**	**13 (4)**
Prefer not to say	4 (5)	3 (3)	3 (3)	11 (11)
**Religious identity**				
Muslim	**5 (2)** **	4 (1)	**4 (1)** *	**13 (4)** ***
Hindu	5 (3)	4 (1)	4 (2)	13 (5)
Others	4 (3)	3 (3)	4 (3)	11 (9)
**Age (years)**				
≤17	5 (2)	**4 (1)** ***	**4 (1)** *	**13 (4)** **
18–25	5 (3)	4 (1)	4 (2)	12.5 (4)
≥26	5 (2)	3 (1)	4 (1)	13 (3)
**Schooling**				
Up to HSC	5 (2)	**4 (1)** *	**4 (1)** *	**13 (4)** *
Honors	5 (3)	4 (2)	4 (2)	12 (4)
Masters and above	5 (3)	4 (1)	4 (2)	13 (4)
**Marital status**				
Never married	5 (2)	**4 (1)** ***	4 (1)	13 (4)
Ever married	5 (2)	4 (1)	4 (2)	12.5 (4)
**Living status**				
With family (including children)	**5 (2)** **	**4 (1)** *	**4 (1)** **	**13 (4)** *
Alone or with friends	5 (3)	3 (2)	4 (3)	12 (6)
**Living location**				
Rural and Suburban	5 (3)	4 (1) ***	4 (2) *	13 (4) **
Urban	5 (2)	**4 (1)**	**4 (1)**	**13 (4)**
**Health status**				
Poor	4 (4) **	3.5 (3) ***	4 (4) ***	11.5 (11) **
Fair	5 (3)	4 (1)	4 (2)	12 (4)
Good	**5 (2)**	3 (1)	4 (2)	13 (4)
Very good	5 (2)	**4 (1)**	**4 (1)**	**13 (3)**
Excellent	5 (3)	4 (1)	4 (1)	13 (4)
**Ever COVID-19 affected**				
No	**5 (2)** *	**4 (1)** ***	**4 (1)** *	**13 (4)** *
Maybe/not sure	5 (3)	4 (1)	4 (2)	12 (4)
Yes	5 (3)	3 (1)	4 (2)	12 (4)
**Media exposure**				
Never	4 (4) *	**3 (3)** *	3 (4) *	11 (9) *
Occasionally/sometimes/often	5 (2)	4 (1)	4 (1)	13 (3)
Always	**5 (3)**	4 (1)	**4 (1)**	**13 (4)**
**Source of information**				
Social media	5 (3) *	4 (1) *	4 (2) *	12 (4) *
YouTube and online news	**5 (2)**	4 (1)	4 (2)	13 (4)
Radio and television	**5 (2)**	**4 (1)**	**4 (1)**	**13 (4)**
**Time spent (hours) on media**				
≤2 h	5 (2) ***	**4 (1)** **	**4 (2)** *	**13 (4)** *
3–4 h	**5 (2)**	4 (1)	4 (2)	13 (4.5)
5 h and above	5 (3)	4 (2)	4 (1)	12 (5)
**Exposure to media compared to pre-COVID-19 situation**				
Decreased	5 (3) ***	3 (2) *	4 (2) *	12 (6) *
About the same	5 (3)	4 (1)	4 (1)	13 (4)
Increased	**5 (2)**	**4 (1)**	**4 (1)**	**13 (4)**

Note: * *p* < 0.001; ** *p* < 0.01; *** *p* < 0.05. Bold and asterisk indicate the probability value < 0.05

**Table 5:** Generalized ordered logistic regression of knowledge, attitude, and practice level with factors associated with Black Fungus.

Factors	Knowledge		Attitude		Practice	
	Moderate and Higher vs. Lower aOR (95%CI)	Higher vs. Lower or Moderate aOR (95%CI)	Moderate and Higher vs. Lower aOR (95%CI)	Higher vs. Lower or Moderate aOR (95%CI)	Moderate and Higher vs. Lower aOR (95%CI)	Higher vs. Lower or Moderate aOR (95%CI)
**Sex Identity**						
Male **(r)**						
Female	**1.49 [1.23, 1.81]**	**1.27 [1.01, 1.60]**	**1.58 [1.30, 1.91]**	**1.41 [1.00, 1.99]**	**1.56 [1.25, 1.95]**	**1.57 [1.29, 1.91]**
Prefer not to say	1.12 [0.43, 2.91]	0.32 [0.04, 2.45]	0.79 [0.30, 2.07]	0.58 [0.07, 4.52]	**0.34 [0.13, 0.92]**	0.69 [0.21, 2.21]
**Religious identity**						
Muslim **(r)**						
Hindu	0.77 [0.53, 1.11]	1.02 [0.66, 1.59]	**1.55 [1.04, 2.29]**	0.79 [0.39, 1.61]	0.77 [0.51, 1.16]	0.92 [0.63, 1.35]
Others	0.57 [0.29, 1.15]	0.55 [0.19, 1.60]	0.64 [0.32, 1.29]	2.09 [0.77, 5.69]	0.67 [0.31, 1.43]	1.02 [0.49, 2.13]
**Age (years**)						
≤17 **(r)**						
18–25	1.18 [0.91, 1.54]	1.10 [0.81, 1.49]	1.21 [0.93, 1.56]	0.80 [0.49, 1.28]	1.20 [0.89, 1.62]	0.93 [0.72, 1.21]
≥26	1.67 [0.87, 3.22]	1.54 [0.67, 3.52]	**2.21 [1.15, 4.24]**	1.14 [0.34, 3.83]	2.02 [0.96, 4.23]	**3.13 [1.55, 6.33]**
**Schooling**						
Up to HSC **(r)**						
Honors	0.82 [0.59, 1.15]	0.92 [0.61, 1.38]	0.74 [0.53, 1.03]	1.39 [0.76, 2.55]	0.90 [0.62, 1.29]	0.79 [0.56, 1.12]
Masters and above	0.75 [0.43, 1.30]	0.91 [0.45, 1.85]	0.74 [0.43, 1.29]	0.79 [0.26, 2.43]	0.84 [0.45, 1.57]	0.61 [0.32, 1.14]
**Marital status**						
Never married **(r)**						
Ever married	0.89 [0.63, 1.25]	0.72 [0.46, 1.11]	**0.64 [0.45, 0.90]**	1.18 [0.64, 2.18]	0.80 [0.55, 1.17]	0.72 [0.50, 1.04]
**Living status**						
With family (including children) **(r)**						
Alone or with friends	0.89 [0.57, 1.42]	**0.45 [0.21, 0.95]**	0.75 [0.46, 1.18]	0.90 [0.37, 2.22]	0.72 [0.45, 1.17]	1.01 [0.61, 1.67]
**Living location**						
Rural and Suburban **(r)**						
Urban	1.03 [0.85, 1.25]	0.91 [0.72, 1.15]	1.24 [1.03, 1.51]	1.24 [0.87, 1.76]	**1.33 [1.07, 1.66]**	1.10 [0.90, 1.34]
**Health status**						
Poor **(r)**						
Fair	0.92 [0.41, 2.06]	1.08 [0.36, 3.26]	0.91 [0.40, 2.06]	1.21 [0.13, 11.01]	0.93 [0.39, 2.2]	0.44 [0.19, 1.05]
Good	1.45 [0.70, 2.99]	1.45 [0.54, 3.91]	1.12 [0.54, 2.34]	2.83 [0.37, 21.59]	1.41 [0.65, 3.07]	0.63 [0.29, 1.35]
Very good	1.58 [0.76, 3.25]	1.25 [0.46, 3.40]	1.18 [0.56, 2.48]	2.23 [0.29, 17.22]	1.53 [0.69, 3.38]	0.70 [0.32, 1.52]
Excellent	1.54 [0.73, 3.23]	1.81 [0.66, 4.96]	0.98 [0.46, 2.06]	2.20 [0.28, 17.09]	1.62 [0.73, 3.61]	0.82 [0.37, 1.79]
**Ever COVID-19 affected**						
No **(r)**						
Maybe/not sure	**0.77 [0.60, 0.99]**	0.86 [0.62, 1.18]	0.92 [0.72, 1.19]	0.90 [0.56, 1.44]	0.87 [0.66, 1.16]	**0.74 [0.57, 0.98]**
Yes	0.79 [0.50, 1.23]	0.74 [0.40, 1.36]	0.65 [0.41, 1.02]	1.14 [0.54, 2.39]	0.82 [0.49, 1.36]	0.63 [0.38, 1.03]
**Media exposure**						
Never **(r)**						
Occasionally/sometimes/often	**1.74 [1.23, 2.46]**	1.46 [0.91, 2.34]	**1.91 [1.35, 2.72]**	0.88 [0.47, 1.65]	**2.75 [1.92, 3.94]**	**1.71 [1.15, 2.53]**
Always	**2.05 [1.41, 2.97]**	**2.11 [1.29, 3.47]**	**2.29 [1.57, 3.33]**	1.19 [0.62, 2.31]	**3.80 [2.57, 5.66]**	**1.75 [1.16, 2.66]**
**Source of information**						
Social media **(r)**						
YouTube and online news	1.22 [0.93, 1.60]	**0.70 [0.49, 0.98]**	1.14 [0.88, 0.50]	**2.39 [1.48, 3.85]**	**1.17 [0.87, 1.58]**	1.19 [0.91, 1.58]
Radio and television	**1.27 [1.03, 1.58]**	0.96 [0.75, 1.24]	**1.34 [1.08, 1.67]**	**1.78 [1.17, 2.72]**	**1.39 [1.08, 1.79]**	**1.44 [1.16, 1.80]**
**Time spent (hours) on media**						
≤Two hours **(r)**						
3–4 h	1.08 [0.86, 1.35]	1.09 [0.84, 1.42]	0.90 [0.72, 1.12]	0.92 [0.63, 1.36]	**0.74 [0.58, 0.95]**	0.95 [0.76, 1.18]
5 h and above	**0.73 [0.54, 0.97]**	1.03 [0.72, 1.47]	1.05 [0.78, 1.41]	0.58 [0.30, 1.11]	**0.57 [0.42, 0.78]**	**0.58 [0.42, 0.80]**
**Exposure to media compared to pre-COVID-19 situation**						
Decreased **(r)**						
About the same	1.03 [0.71, 1.49]	0.71 [0.45, 1.11]	1.18 [0.81, 1.72]	**2.39 [1.00, 5.72]**	**1.57 [1.05, 2.33]**	1.44 [0.95, 2.18]
Increased	1.27 [0.89, 1.81]	0.81 [0.53, 1.23]	**1.49 [1.04, 2.12]**	2.14 [0.91, 5.01]	**1.57 [1.07, 2.27]**	**1.56 [1.06, 2.31]**
LR χ2	**131.92**		**154.53**		**224.06**	

Values in bold are significantly different at 5% level of significance (*p* < 0.05); aOR: adjusted odds ratio; **(r)**: reference group; LR: likelihood ratio; χ2 = Chi-squared.

## Data Availability

Data can be found at the following link: https://dataverse.harvard.edu/dataset.xhtml?persistentId=doi:10.7910/DVN/LRZUC9&faces-redirect=true.

## References

[1] Lai C.-C., Shih T.-P., Ko W.-C., Tang H.-J., Hsueh P.-R. (2020). Severe acute respiratory syndrome coronavirus 2 (SARS-CoV-2) and coronavirus disease-2019 (COVID-19): The epidemic and the challenges. Int. J. Antimicrob. Agents.

[2] Cucinotta D., Vanelli M. (2020). WHO declares COVID-19 a pandemic. Acta Bio. Med. Atenei Parm..

[3] World Health Organization (2022). Weekly Operational Update on COVID-19-15 February 2022.

[4] World Health Organization (2022). COVID-19 Bangladesh Situation Reports: Situation Report 24 January 2022.

[5] Ahmed M.Z., Ahmed O., Aibao Z., Hanbin S., Siyu L., Ahmad A. (2020). Epidemic of COVID-19 in China and associated psychological problems. Asian J. Psychiatr..

[6] Chen L., Yuan X. (2020). China’s ongoing battle against the coronavirus: Why did the lockdown strategy work well?. Socio-Ecol. Pract. Res..

[7] Akter R., Akter M., Hossain M.T., Ahsan M.N. (2021). Socio-environmental factors affecting mental health of people during COVID-19 in coastal urban areas of Bangladesh. Environmental Resilience and Transformation in Times of COVID-19.

[8] Fazel M., Wheeler J., Danesh J. (2005). Prevalence of serious mental disorder in 7000 refugees resettled in western countries: A systematic review. Lancet.

[9] Zheng W. (2020). Mental health and a novel coronavirus (2019-nCoV) in China. J. Affect. Disord..

[10] Patel R., Baker S.S., Liu W., Desai S., Alkhouri R., Kozielski R., Mastrandrea L., Sarfraz A., Cai W., Vlassara H. (2012). Effect of dietary advanced glycation end products on mouse liver. PLoS ONE.

[11] Vickers N.J. (2017). Animal communication: When i’m calling you, will you answer too?. Curr. Biol..

[12] Shovo T.-E.-A., Ahammed B., Khan B., Jahan N., Shohel T.A., Hossain T., Islam N. (2021). Determinants of Generalized Anxiety, Depression, and Subjective Sleep Quality among University Students during COVID-19 Pandemic in Bangladesh. Dr. Sulaiman Al Habib Med. J..

[13] Hossain M.T., Islam M.A., Jahan N., Nahar M.T., Sarker M.J.A., Rahman M.M., Deeba F., Hoque K.E., Aktar R., Islam M.M. (2022). Mental Health Status of Teachers During the Second Wave of the COVID-19 Pandemic: A Web-Based Study in Bangladesh. Front Psychiatry.

[14] Gao J., Zheng P., Jia Y., Chen H., Mao Y., Chen S., Wang Y., Fu H., Dai J. (2020). Mental health problems and social media exposure during COVID-19 outbreak. PLoS ONE.

[15] Hossain M.T., Ahammed B., Chanda S.K., Jahan N., Ela M.Z., Islam M.N. (2020). Social and electronic media exposure and generalized anxiety disorder among people during COVID-19 outbreak in Bangladesh: A preliminary observation. PLoS ONE.

[16] Hammad M.A., Alqarni T.M. (2021). Psychosocial effects of social media on the Saudi society during the Coronavirus Disease 2019 pandemic: A cross-sectional study. PLoS ONE.

[17] Biswas P.R., Ahammed B., Rahman M.S., Nirob B.M., Hossain M.T. (2022). Prevalence and determinants of internet addiction among adults during the COVID-19 pandemic in Bangladesh: An online cross-sectional study. Heliyon.

[18] Raut A., Huy N.T. (2021). Rising incidence of mucormycosis in patients with COVID-19: Another challenge for India amidst the second wave?. Lancet Respir. Med..

[19] Kabir H., Hasan M.K., Rahman M., Akter S., Chowdhury G.I., Bhuya T.R., Mitra D.K. (2021). Black fungus or mucormycosis: A cross-sectional knowledge assessment among the Bangladeshi health care workers during COVID-19 pandemic. PsyArXiv.

[20] Chander J. (2017). Textbook of Medical Mycology.

[21] Skiada A., Pavleas I., Drogari-Apiranthitou M. (2020). Epidemiology and diagnosis of mucormycosis: An update. J. Fungi.

[22] Ishani Ganguli M., Katrina Armstrong M. (2015). Ebola Risk and Preparedness: A National Survey of Internists. J. Gen. Intern. Med..

[23] Sharma N.C. (2021). India Reports 40,854 Cases of Black Fungus So Far. MINT.

[24] Ray S. (2021). India Declares ‘Black Fungus’ Epidemic As Infections Rise Among COVID-19 Patients. Forbes.

[25] Rahman F.I., Islam M.R., Bhuiyan M.A. (2021). Mucormycosis or black fungus infection is a new scare in South Asian countries during the COVID-19 pandemic: Associated risk factors and preventive measures. J. Med. Virol..

[26] Kamruzzaman M. (2021). Bangladesh Detects 2 Cases of Black Fungus in Capital.

[27] SBS A. (2021). Rare ‘Black Fungus,’ mucormycosis, infects thousands of COVID-19 survivors in India. Wall Street Journal.

[28] Daria S., Asaduzzaman M., Shahriar M., Islam M.R. (2021). The massive attack of COVID-19 in India is a big concern for Bangladesh: The key focus should be given on the interconnection between the countries. Int. J. Health Plan. Manag..

[29] Biswas S. (2021). Mucormycosis: The ‘black fungus’ maiming COVID patients in India. BBC News.

[30] National Organization for Rare Disorders (2018). Rare Disease Database: Mucormycosis. https://rarediseases.org/rare-diseases/Mucormycosis/.

[31] Jha R.K. (2021). Explained: Why There Is Shortage of Black Fungus Drug in India.

[32] Spellberg B., Edwards Jr J., Ibrahim A. (2005). Novel perspectives on mucormycosis: Pathophysiology, presentation, and management. Clin. Microbiol. Rev..

[33] Ahmadikia K., Hashemi S.J., Khodavaisy S., Getso M.I., Alijani N., Badali H., Mirhendi H., Salehi M., Tabari A., Mohammadi Ardehali M. (2021). The double-edged sword of systemic corticosteroid therapy in viral pneumonia: A case report and comparative review of influenza-associated mucormycosis versus COVID-19 associated mucormycosis. Mycoses.

[34] Sen M., Honavar S.G., Sharma N., Sachdev M.S. (2021). COVID-19 and eye: A review of ophthalmic manifestations of COVID-19. Indian J. Ophthalmol..

[35] Gizaw G.D., Alemu Z.A., Kibret K.T. (2015). Assessment of knowledge and practice of health workers towards tuberculosis infection control and associated factors in public health facilities of Addis Ababa, Ethiopia: A cross-sectional study. Arch. Public Health.

[36] Watkins J. (2020). Preventing a COVID-19 pandemic. BMJ.

[37] Wake A.D. (2020). Knowledge, attitude, practice, and associated factors regarding the novel coronavirus disease 2019 (COVID-19) pandemic. Infect. Drug Resist..

[38] Oladele R., Otu A.A., Olubamwo O., Makanjuola O.B., Ochang E.A., Ejembi J., Irurhe N., Ajanaku I., Ekundayo H.A., Olayinka A. (2020). Evaluation of knowledge and awareness of invasive fungal infections amongst resident doctors in Nigeria. Pan Afr. Med. J..

[39] Hasan M.K., Kabir H., Rahman M., Roy A.K., Akter S., Mitra D.K. (2021). Association between insomnia and mucormycosis fear among the Bangladeshi healthcare workers: A cross-sectional study. J. Affect. Disord. Rep..

[40] Ahammed B., Jahan N., Seddeque A., Hossain M.T., Khan B., Mamun M.A., Islam M.N. (2021). Exploring the association between mental health and subjective sleep quality during the COVID-19 pandemic among Bangladeshi university students. Heliyon.

[41] Islam M.A., Barna S.D., Raihan H., Khan M.N.A., Hossain M.T. (2020). Depression and anxiety among university students during the COVID-19 pandemic in Bangladesh: A web-based cross-sectional survey. PLoS ONE.

[42] Obenauer J., Rübsamen N., Garsevanidze E., Karch A., Mikolajczyk R.T. (2018). Changes in risk perceptions during the 2014 Ebola virus disease epidemic: Results of two consecutive surveys among the general population in Lower Saxony, Germany. BMC Public Health.

[43] Ferdous M.Z., Islam M.S., Sikder M.T., Mosaddek A.S.M., Zegarra-Valdivia J., Gozal D. (2020). Knowledge, attitude, and practice regarding COVID-19 outbreak in Bangladesh: An online-based cross-sectional study. PLoS ONE.

[44] Kundu S., Al Banna M., Sayeed A., Begum M.R., Brazendale K., Hasan M.T., Habiba S.J., Abid M.T., Khan M., Chowdhury S. (2021). Knowledge, attitudes, and preventive practices toward the COVID-19 pandemic: An online survey among Bangladeshi residents. J. Public Health.

[45] Hayat K., Rosenthal M., Xu S., Arshed M., Li P., Zhai P., Desalegn G.K., Fang Y. (2020). View of Pakistani residents toward coronavirus disease (COVID-19) during a rapid outbreak: A rapid online survey. Int. J. Environ. Res. Public Health.

[46] Rugarabamu S., Ibrahim M., Byanaku A. (2020). Knowledge, attitudes, and practices (KAP) towards COVID-19: A quick online cross-sectional survey among Tanzanian residents. MedRxiv.

[47] Rahman M.M., Khan S.J., Sakib M.S., Halim M.A., Rahman M.M., Asikunnaby, Jhinuk J.M. (2021). COVID-19 responses among university students of Bangladesh: Assessment of status and individual view toward COVID-19. J. Hum. Behav. Soc. Environ..

[48] Zhong B.-L., Luo W., Li H.-M., Zhang Q.-Q., Liu X.-G., Li W.-T., Li Y. (2020). Knowledge, attitudes, and practices towards COVID-19 among Chinese residents during the rapid rise period of the COVID-19 outbreak: A quick online cross-sectional survey. Int. J. Biol. Sci..

[49] Williams R. (2006). Generalized ordered logit/partial proportional odds models for ordinal dependent variables. Stata J..

[50] Banik R., Rahman M., Sikder M.T., Rahman Q.M., Pranta M.U.R. (2021). Knowledge, attitudes, and practices related to the COVID-19 pandemic among Bangladeshi youth: A web-based cross-sectional analysis. J. Public Health.

[51] Farhana K., Mannan K. (2020). Knowledge and Perception Towards Novel Coronavirus (COVID 19) in Bangladesh (SSRN Scholarly Paper ID 3576523). Soc. Sci. Res. Netw..

[52] Fernandes B., Biswas U.N., Mansukhani R.T., Casarín A.V., Essau C.A. (2020). The impact of COVID-19 lockdown on internet use and escapism in adolescents. Rev. De Psicol. Clínica Con Niños Y Adolesc..

[53] Hossain I. Mobile Operators Struggling to Cope with Rise in Data Consumption. Finance Express. p. 6. https://thefinancialexpress.com.bd/trade/mobile-operators-struggling-to-cope-with-rise-in-data-consumption-1587611418.

[54] Zhao N., Zhou G. (2021). COVID-19 stress and addictive social media use (SMU): Mediating role of active use and social media flow. Front. Psychiatry.

[55] Clements J.M. (2020). Knowledge and behaviors toward COVID-19 among US residents during the early days of the pandemic: Cross-sectional online questionnaire. JMIR Public Health Surveill..

[56] Almohammed O.A., Aldwihi L.A., Alragas A.M., Almoteer A.I., Gopalakrishnan S., Alqahtani N.M. (2021). Knowledge, attitude, and practices associated with COVID-19 among healthcare workers in hospitals: A cross-sectional study in Saudi Arabia. Front. Public Health.

[57] Wolf M.S., Serper M., Opsasnick L., O’Conor R.M., Curtis L., Benavente J.Y., Wismer G., Batio S., Eifler M., Zheng P. (2020). Awareness, attitudes, and actions related to COVID-19 among adults with chronic conditions at the onset of the US outbreak: A cross-sectional survey. Ann. Intern. Med..

[58] Hong H.-L., Lee Y.-M., Kim T., Lee J.-Y., Chung Y.-S., Kim M.-N., Kim S.-H., Choi S.-H., Kim Y.S., Woo J.H. (2013). Risk factors for mortality in patients with invasive mucormycosis. Infect. Chemother..

[59] Dyer O. (2021). COVID-19: India sees record deaths as “black fungus” spreads fear. BMJ.

[60] Kassie B.A., Adane A., Tilahun Y.T., Kassahun E.A., Ayele A.S., Belew A.K. (2020). Knowledge and attitude towards COVID-19 and associated factors among health care providers in Northwest Ethiopia. PLoS ONE.

[61] Endriyas M., Kawza A., Alano A., Hussen M., Mekonnen E., Samuel T., Shiferaw M., Ayele S., Kelaye T., Misganaw T. (2021). Knowledge and attitude towards COVID-19 and its prevention in selected ten towns of SNNP Region, Ethiopia: Cross-sectional survey. PLoS ONE.

[62] Giao H., Thi N., Han N., Khanh T.V., Ngan V., Tam V. (2020). Knowledge and attitude toward COVID-19 among healthcare workers at Knowledge and attitude toward COVID-19 among healthcare workers at District 2 Hospital, Ho Chi Minh City. Asian Pac. J. Trop. Med..

[63] Azlan A.A., Hamzah M.R., Sern T.J., Ayub S.H., Mohamad E. (2020). Public knowledge, attitudes and practices towards COVID-19: A cross-sectional study in Malaysia. PLoS ONE.

[64] Saqlain M., Munir M.M., Rehman S.U., Gulzar A., Naz S., Ahmed Z., Tahir A.H., Mashhood M. (2020). Knowledge, attitude, practice and perceived barriers among healthcare workers regarding COVID-19: A cross-sectional survey from Pakistan. J. Hosp. Infect..

[65] Srichan P., Apidechkul T., Tamornpark R., Yeemard F., Khunthason S., Kitchanapaiboon S., Wongnuch P., Wongphaet A., Upala P. (2020). Knowledge, attitudes and preparedness to respond to COVID-19 among the border population of northern Thailand in the early period of the pandemic: A crosssectional study. WHO South-East Asia J. Public Health.

[66] Rahman A., Sathi N.J. (2020). Knowledge, Attitude, and Preventive Practices toward COVID-19 among Bangladeshi Internet Users. Electron. J. Gen. Med..

[67] Islam S., Emran G.I., Rahman E., Banik R., Sikder T., Smith L., Hossain S. (2021). Knowledge, attitudes and practices associated with the COVID-19 among slum dwellers resided in Dhaka City: A Bangladeshi interview-based survey. J. Public Health.

[68] Leung G.M., Ho L.-M., Chan S.K., Ho S.-Y., Bacon-Shone J., Choy R.Y., Hedley A.J., Lam T.-H., Fielding R. (2005). Longitudinal assessment of community psychobehavioral responses during and after the 2003 outbreak of severe acute respiratory syndrome in Hong Kong. Clin. Infect. Dis..

[69] Peng Y., Pei C., Zheng Y., Wang J., Zhang K., Zheng Z., Zhu P. (2020). A cross-sectional survey of knowledge, attitude and practice associated with COVID-19 among undergraduate students in China. BMC Public Health.

